# Would changing the selection process for GP trainees stem the workforce crisis? A cohort study using multiple-imputation and simulation

**DOI:** 10.1186/s12909-018-1160-z

**Published:** 2018-04-27

**Authors:** Celia Taylor, I. C. McManus, Ian Davison

**Affiliations:** 1 Division of Health Sciences, Warwick Medical School, Coventry, CV4 7AL UK; 2 Department of Psychology, University College London, Gower Street, WC1E 6BT, London, UK; 3 School of Education, University of Birmingham, Edgbaston, B15 2TT UK

**Keywords:** General practice, Recruitment, Selection, Training, Multiple imputation

## Abstract

**Background:**

There is currently a shortage of qualified GPs in the UK and not all of the training posts available each year are filled. Changing the way in which GP trainees are selected could help increase the training post fill rate and the number of new entrants to the GP Register. The aim of this study was to model the impact of changing the selection process for GP training on the number of trainees obtaining GP Registration, either with or without extensions.

**Method:**

This was a cohort study using UK applications for GP training in 2011–14. Application data were linked using GMC numbers to training outcome data where available, and imputed using multiple imputation where missing. The number of trainees appointed and GP Registrations within three and five years’ full-time-equivalent were estimated for four different selection processes.

**Results:**

The cut scores used in the actual 2015 selection process makes it impossible to fill all training posts. Random selection is the worst option, but the difference between this and other processes modelled falls as more trainees are selected. There are large marginal effects on outcomes: those with the highest selection scores are more likely to obtain GP Registration than those with the lowest scores.

**Conclusions:**

Changing the selection process alone would have a small impact on the number of GP Registrations; reducing/removing cut scores would have a much larger impact. This would also increase the number of trainees requiring extensions and being released from training which would have adverse consequences for the profession.

## Background

The Centre for Workforce Intelligence’s review of the English General Practitioner (GP) workforce concluded that “the current level of GPs being trained is inadequate and likely to lead to a major workforce demand-supply imbalance by 2020 unless action is taken” [1], p. 5. The review therefore recommended “a substantial increase in GP training numbers” [1], p. 5. There are currently around 3900 new GP training posts available across the UK each year [2–4]. Any increase in the number of GP training posts available will only increase GP supply if the additional, or *marginal*, posts are filled *and* the marginally recruited trainees successfully complete training and obtain GP Registration.

The training of GPs currently involves a three-year programme in which trainees undertake a combination of hospital- and general practice-based posts. Before the end of that time they sit the Membership examination of the Royal College of General Practitioners (MRCGP), which has two parts, the Applied Knowledge Test (AKT) and the Clinical Skills Assessment (CSA). If these are passed, and in-training Work-place Based Assessments (WPBAs) have been satisfactory, the doctor can apply for entry onto the General Medical Council’s (GMC) GP Register (i.e. obtain GP Registration), which allows independent practice as a GP.

Whether all GP training posts are filled depends on *recruitment* and *selection* processes. Whether GP trainees successfully complete training depends on their *suitability* for training and the *quality* of the training program. Recruitment is about getting suitable doctors to apply for GP training, and, where offered, to accept a GP training post. However, recruitment is clearly becoming increasingly challenging: the number of doctors applying for GP training in Round 1 fell by 22% from 6200 in 2009 to 4863 in 2016 [6, 7]. Selection firstly seeks to identify applicants who will ultimately obtain GP Registration (i.e. are considered suitable for training) and secondly, where the number of suitable applicants exceeds the number of GP training posts, to rank applicants and fill posts with those considered most suitable. The current GP trainee selection process in the UK involves three stages, an eligibility check (Stage 1), the Multi-specialty Recruitment Assessment, which comprises computer-based assessments of Clinical Problem Solving and Professional Dilemmas (Stage 2) and, for those achieving set cut scores on these assessments, a Selection Centre with three face-to-face simulations and a written assessment, during which applicants’ competency on various attributes is compared to that considered to be required for training (Stage 3) [4]. Even with the decline in applicant numbers, all 3900 posts available in 2016 could have been filled. However, an insufficient number were considered suitable and there was a decline in the overall *fill rate* from 96% in 2009 (*N* = 3213/3344 training posts) to 90% in 2016 (*N* = 3520/3896 training posts) [6].

A number of new initiatives were put in place from the 2016/17 recruitment round to help increase recruitment numbers [11] although the effect of these initiatives on applicant numbers and the fill rate is not yet known. The cut scores applied during selection (to determine suitability for training) could also be reduced to increase the number of applicants selected into training. However this may disproportionately increase the number of GP trainees requiring extended training time and/or failing to obtain GP Registration. The ensuing consequences include increased financial costs of additional training, emotional costs to the trainees themselves and those organising and delivering training, a threat to the reputation of General Practice, and potential threats to patient safety and patient health if the marginal trainees cannot provide care of a sufficient quality.

This paper uses a unique dataset to quantify the risk of marginally selected trainees failing and model the potential impact of changing the selection process for GP training on the number of trainees obtaining GP Registration, either with or without extensions.

## Methods

### Data

Selection data (as detailed in Table 1) for all applications to UK GP training between 2011 and 2014 were provided by the GP National Recruitment Office (GPNRO). Any applications failing Stage 1 or withdrawing prior to taking Stage 2 were excluded from the analysis, meaning only applications with a Stage 2 score were included. Performance data (up to 27 August 2015) for all doctors taking up GP training posts between August 2011 and August 2014 were provided by the GMC, Health Education bodies in the UK and the RCGP (Table 1). Selection and training performance data were linked using GMC numbers. This meant that a small number of applications missing a GMC number were excluded from the analysis. Where a doctor had applied on multiple occasions, training performance data were linked to their successful application. The unit of analysis was therefore the *application* for GP training, rather than the doctor applying.

**Table 1:** Variables included in the selection and performance datasets and the multiple imputation

Selection dataset: GMC number, application Round, selection scores and progression through each Stage of the selection process, applicant decisions (withdrawal from the selection process and whether a post offer was accepted or declined) and personal characteristics (including gender, ethnicity and country of primary medical qualification).
Performance dataset: GMC number, date training commenced, Annual Review of Competence Progression (ARCP) outcomes (as detailed in *The Gold Guide* [12]), indicators of less than full time (LTFT)/Out of Programme (OOP) status, MRCGP examination performance on the AKT and CSA including date taken and score achieved relative to the pass mark and date of GP Registration (if obtained).
Used in multiple imputation as part of the prediction algorithm (available for all applications): Round of application (those applying in Round 3 in 2014 were re-coded into Round 2; data for 2010 were not available), Stage 2 scores, gender, ethnicity (coded as white/black and ethnic minority), and country of primary medical qualification (coded as UK/non-UK).
Imputed for each application where actual data were missing: withdrawal from selection process, Stage 3 total score (the sum of the competency scores across the three scenarios and written assessment), offer accept/decline, LTFT, OOP, ARCP Outcome 4, and FTE-equivalent actual time to GP Registration if no ARCP Outcome 4.

We created a variable which assessed actual time to GP Registration, which, in order to avoid penalising doctors spending time OOP or working LTFT, was adjusted to reflect the full-time equivalent (FTE) equivalent duration. Any time spent OOP was subtracted and the remainder reduced by a factor of 1.67 for every year spent LTFT. This factor is equivalent to a LTFT doctor working at 60% FTE; no data were available on actual FTE so we had to assume a common value for all.

### Multiple imputation of missing data

The only variable available for every application was Stage 2 scores achieved. A recurrent problem with evaluating any selection process is that final outcomes can only be known for those who are selected, whereas evaluation wishes to assess what would have happened were rejected candidates actually selected (for review see McManus and colleagues [13]). We only had complete data for applications where the doctor was offered and accepted a GP training post and either obtained GP Registration or who had an ARCP Outcome 4: Released from training [12]. This problem can be addressed by treating the data as a missing values problem, using the expectation-maximisation algorithm, or, preferably – and the approach taken here - multiple imputation, since this allows repeated imputations to assess the variability of estimated values [14] .

Multiple imputation was undertaken using SPSS v.22, using a ‘fully conditional’ algorithm, in which each variable in turn is taken as the dependent variable, using all other variables as predictors [15] (Table 1). The algorithm automatically takes restriction in range (i.e. that those selected have higher selection scores than those not selected) into account. Ten separate imputations were undertaken to give an adequate sense of variability without imposing major computational constraints.

### Analysis of multiple imputation results

Analysis was undertaken using Stata v11 and the results are reported as annual means to enable interpretation against annual recruitment targets. Where GP Registration was actually or imputed to have been obtained, the FTE-equivalent actual time to GP Registration was compared to the expected time to GP Registration, which was set at three years plus a two month grace period to allow for any delays in processing applications. We coded each application across two dichotomous outcomes: whether GP Registration was or would be obtained (1) within three or (2) within five years FTE training time.

We considered four potential selection processes (Table 2). For each imputation, we identified the applications that would have resulted in a filled training post using each selection process for seven annual recruitment targets: 1000, 1500, 2000, 2500, 3000, 3500 and 4000. We then found the arithmetic mean and standard deviation of the number of entrants to the GP Register at three and five years for each selection process/recruitment target combination across the ten imputations. The standard deviation of the imputed estimates is, in effect, the standard error, and, if required, the 2.5th and 97.5th percentiles of the estimates could be used as approximate bounds of a 95% confidence interval.

**Table 2:** Selection processes modelled

Random selection: The baseline condition in which trainees are selected at random from Round 1 applications until all training posts are filled.
2015 selection process: The actual selection process used in 2015 in which applications in both Rounds 1 and 2 had to achieve a cut score of 181 on each Stage 2 test and obtain a final Stage 3 outcome of “demonstrated competency for training” to be offered a training post. Note that the constraints imposed by these cut scores meant that in practice it was not possible for more than 3000 training posts to be filled.
Stage 2 selection only: Round 1 applications are sorted on their total Stage 2 scores and trainees selected from the highest to lowest scores until all posts are filled. No cut score is applied.
Stage 3 selection only: Round 1 applications are sorted on their Stage 3 total scores and trainees selected from the highest to lowest scores until all posts are filled. No cut score is applied.

Applications withdrawing from the selection process or declining offers are excluded from all processes. A second selection round (Round 2) is only required for the 2015 selection process: for all other processes, all training posts would be filled during Round 1

## Results

Table 3 provides data on the numbers of applications included in the analysis, as well as the maximum number of GP trainees who could have been appointed had no applications been rejected at Stages 2 or 3 of the selection process.

**Table 3:** Application numbers, 2011 to 2014 combined

	Round 1	Round 2*
Applications included (with GMC number, passing Stage 1 and not withdrawing prior to Stage 2)	20,782 (mean 5196 per year)	4578 (mean 1145 per year)
Of which did, or were imputed to have, withdrawn before Stage 3 or declined offer of a post (mean across the 10 imputations)	4174 (mean 1044 per year)	778 (mean 195 per year)
Maximum number of posts that could have been filled (Applications included minus withdrawals and declines)	16,608 (mean 4152 per year)	3800 (mean 950 per year)

*These data are only used when modelling the 2015 selection process (see Table 1); for all other processes modelled all posts are filled in Round 1

**Fig. 1:**
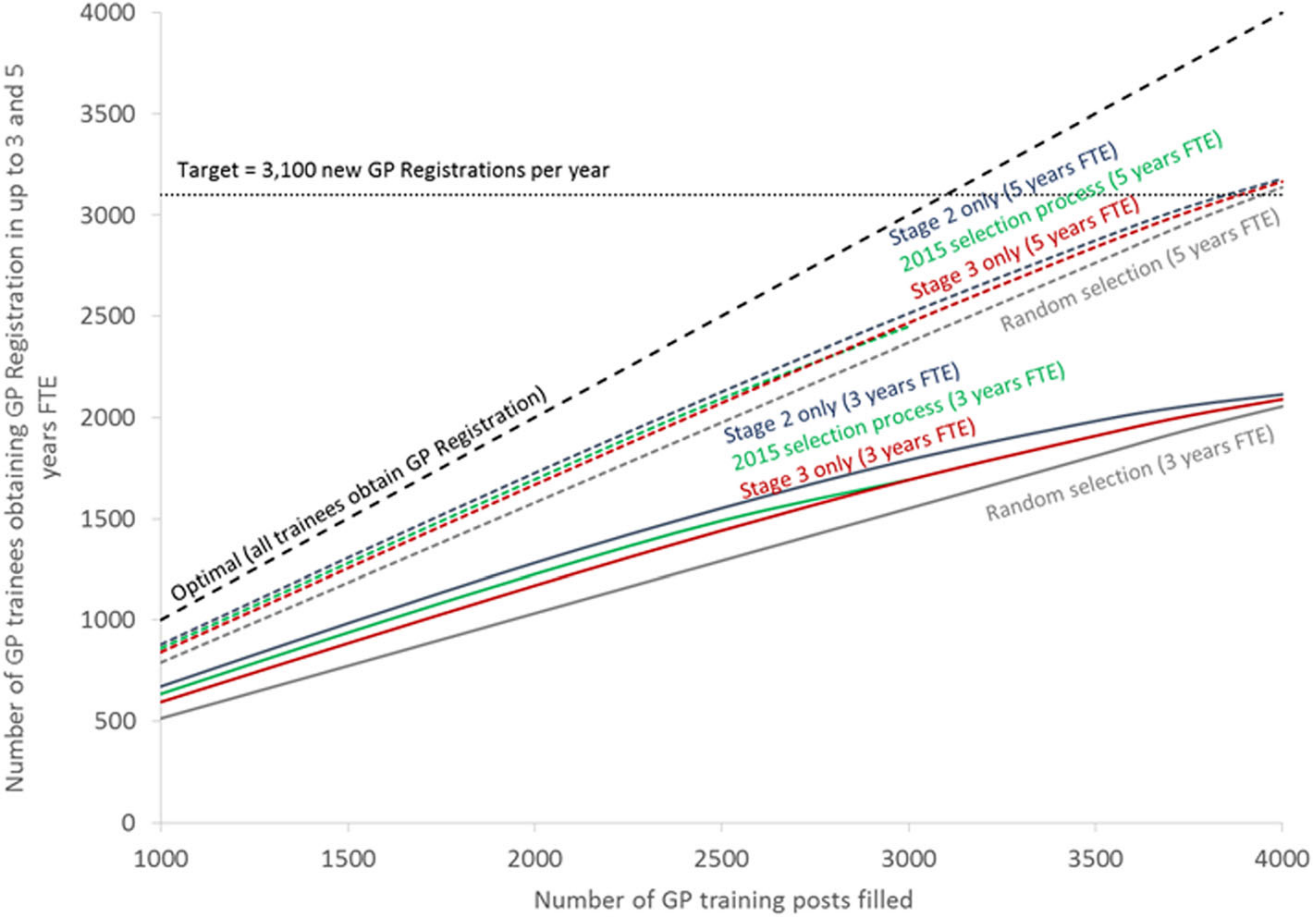
The imputed relationship between the number of GP training posts filled and the number of GP Registrations within 3 and 5 years FTE

Figure 1 plots the full results of the analysis, with the data used to plot these lines in Table 4. We use as the target number of GP Registrations a published estimate of the required number of GPs beginning to practice in the UK of 3100 per year [5]. The standard deviations of each set of ten individual multiple imputations are relatively low, which suggest our results are sufficiently precise for our purposes. Figure 1 can be used to illustrate five key results.

**Table 4:** Annual number of those recruited achieving GP Registration within 3 and 5 years FTE with each number of posts filled and selection process (mean (SD) across the 10 multiple imputations), based on 2011 to 2014 applications

Posts filled	2015 selection process		Random selection		Stage 2 only		Stage 3 only	
	3 years	5 years	3 years	5 years	3 years	5 years	3 years	5 years
1,000	636 (15)	861 (8.0)	515 (15)	791 (13)	673 (14)	881 (5.9)	596 (12)	842 (8.4)
1,500	939 (17)	1,281 (8.9)	774 (22)	1,185 (17)	985 (17)	1,308 (7.2)	886 (17)	1,256 (11)
2,000	1,227 (20)	1,694 (9.7)	1,034 (27)	1,580 (22)	1,283 (21)	1,726 (8.7)	1,171 (19)	1,668 (11)
2,500	1,491 (18)	2,094 (12)	1,293 (30)	1,976 (25)	1,553 (21)	2,217 (10)	1,442 (20)	2,072 (13)
3,000	1,693 (21)	2,448 (14)	1,551 (31)	2,370 (28)	1,791 (23)	2,512 (15)	1,692 (23)	2,466 (15)
3,500	N/A	N/A	1,811 (36)	2,764 (30)	1,983 (28)	2,873 (21)	1,910 (26)	2,841 (19)
4,000	N/A	N/A	2,056 (38)	3,138 (39)	2,115 (32)	3,178 (37)	2,091 (32)	3,615 (34)

N/A Not possible for the required number of doctors to obtain GP Registration

Firstly, although in an optimal selection process all those selected would obtain GP Registration, resulting in the dashed diagonal line with a gradient of 1 at the top of the figure, in reality the lines for all of the selection processes modelled have a gradient of less than 1 and are therefore below the optimal selection process line. This implies that, regardless of which selection process is used, some trainees will require extensions to training and some will not enter the GP Register within five years. As would be expected, the worst selection process is random selection, with 52% of those selected obtaining GP Registration within three years and 79% within five years, regardless of the number selected.

Secondly, the gradients of all the lines, except that for random selection, fall slightly as the number of training posts filled increases, as a result of diminishing marginal returns to selection. When relatively few training posts are filled, those selected are more likely to obtain GP Registration compared to when more posts are filled. If the 1000 top-ranked applicants are selected using the 2015 selection process, 86% would be expected to obtain GP Registration within five years, of whom 26% would require an extension. If the top 3000 applicants are selected, the overall proportion who would be expected to obtain GP Registration within five years falls slightly to 82%, of whom 31% would require an extension. However of the last or marginal 500 (i.e. those ranked 2501 to 3000), only 71% would obtain GP Registration within five years, with 43% of requiring an extension. The implication is that as the number of posts filled increases, the relative effectiveness of any selection process declines in relation to random selection and the curves in Fig. 1 begin to converge to that of random selection. With 3000 posts filled, the 2015 selection process only provides around 80 more trainees obtaining GP Registration within five years than would have been achieved with random selection, an increase of approximately 3%.

Thirdly, because cut scores are used, the 2015 selection process could not fill more than 3000 training posts (hence the lines are truncated here), so the target of 3100 new GPs per year could never be achieved without recruiting qualified GPs from overseas. To meet this target using the most effective selection process described in this study which is selection based on Stage 2 scores only, around 3860 posts would need to be filled (just under the number that are currently available), and it would still take five years for 3100 trainees to obtain GP Registration.

Fourthly, using any selection process reduces the number of trainees requiring an extension compared to the use of random selection. For example, with 3000 posts filled, around 60 fewer extensions would be required by using the 2015 process compared with random selection, a reduction of approximately 8%. This can be seen in Fig. 1 as, for any number of GP training posts filled, the vertical distance between the three year (solid) and five year (dashed) lines is larger for random selection than for any other individual process.

Finally, while the selection processes modelled vary in their effectiveness, the overall differences between them are fairly small, particularly as the number of posts filled increases. Using Stage 2 scores only rather than the 2015 selection process increases the number of trainees obtaining GP Registration within five years by around 60 (from 2448 to 2512) if 3000 training posts are filled, an increase of approximately 2.5%.

## Discussion

### Summary

Changing the selection process alone would have a relatively small, although perhaps useful effect on the number of trainees entering the GP Register. Of the selection processes modelled in this study, using Stage 2 scores only would be the most effective selection process. A significant increase in the number of trainees obtaining GP Registration would require a reduction in the cut scores used during selection and hence the recruitment of more trainees. However, the advantages of doing so must be weighed against the disadvantages. If 4000 posts are filled using Stage 2 scores only, rather than the 3000 using the current selection process, an additional 730 GPs would enter the GP Register within five years. However, there would also be an additional 300 training extensions that must be funded and supported by Deaneries, and an additional 270 trainees who would be released from training, with trainees in these groups at somewhat greater risk of causing patient harm.

### Strengths and limitations

The multiple imputation process produced valid and reliable results that were consistent across the ten imputations performed [17]. We had access to the entire population of applications for GP training for four years, as well as performance data, and were able to match doctors within the various datasets for the vast majority of those selected. Nevertheless, linking datasets is never without its difficulties and, despite a thorough ‘clean’ of the data, a very small number of errors may remain.

The analysis reported here was undertaken using a UK perspective and assumed that selected trainees would be sufficiently mobile to fill available posts in all regions, which may not be the case in practice, as there are clear regional differences in fill rates [6]. We considered all trainees requiring extensions as a single group, but recognise that a six month extension has different consequences to a two year extension. However, we did not find a significant bias when comparing the effectiveness of selection processes in terms of mean extension length [17]. Finally, we have not costed any of the selection processes (or their consequences) in money terms; although Stage 3 is more expensive than Stage 2 and the 2015 process is particularly expensive since two Rounds of selection are required. The financial impact on Deaneries and patients of increasing recruitment numbers on the number of extensions required and the number of trainees being released from their training programmes needs to be considered carefully. A three year GP training programme costs approximately £210,000 per trainee [18], with a one year extension costing more than one-third of this given the administrative burden, additional training provision and extra ARCP required for the trainee.

### Comparison with existing literature

To our knowledge, this is the first study to use multiple imputation to model the consequences of using different selection processes. It builds on existing work which evaluates the validity and reliability of selection processes for specialty training [8–10, 16] by considering the final outcome of the selection process – the number of GPs entering the GP Register.

### Implications for research and/or practice

While the Stage 2 only selection process was the most effective, its use in practice requires stakeholder consultation, since its effectiveness and cost savings in comparison to Stage 3 must be weighed against the acceptability and educational impact of excluding the face-to-face component. However, the GP selection process now includes a “Direct Pathway” from Stage 2 to receiving an offer for the highest scoring applicants [11], so that there appears to be a shift away from the belief that a face-to-face assessment is critical for all applicants. There may also be unintended consequences of only using Stage 2 for selection, such as increasing applications from those who consider themselves unlikely to succeed at Stage 3; the results presented here are only valid if the composition of the applicant body does not change. The modelling in this paper only considers those who have chosen to apply for GP training, whereas a broader analysis would model the entire cohort of UK and international doctors who are applying for any form of specialty training.

The analysis undertaken here could be repeated for other possible approaches to GP selection, for example applying different weights to the Stage 2 and 3 assessments, but given the closeness of the lines in Fig. 1 it seems unlikely that any such fine-tuning would have a large benefit. Further work to help quantify the patient health consequences of different selection methods such as reducing cut scores would be useful. Such work could draw on studies undertaken overseas, such as that examining the relationship between licensing examination scores and quality of care amongst international medical graduates working in the US [19]. In addition, since changing the selection process alone is unlikely to meet the future demand for GPs, measures designed to enhance recruitment and retention are also essential [1, 3].

While all specialties should benefit from a recent decision by the UK Government to increase the number of medical school places by 1500 per year from 2018 entry [20], any additional entrants would not start GP training until 2025 at the earliest, so that is certainly not a fast-acting solution. There also has to be a concern that the new entrants may not be as well qualified as those currently entering medical school, as the pool of entrants is widened, and those entrants may as a result have higher failure rates at undergraduate and postgraduate [13, 17]. Efforts are therefore needed to attract existing as well as future medical students into GP, which must include tackling the “perilously low morale” of current GPs that can only be a disincentive for those making specialty choice decisions [21].

## Conclusions

Changing the selection process alone would have a small impact on the number of GP Registrations; reducing/removing cut scores would have a much larger impact. This would also increase the number of trainees requiring extensions and being released from training which would have adverse consequences for the profession.

## Abbreviations

AKT: Applied Knowledge Test
ARCP: Annual Review of Competence Progression
CSA: Clinical Skills Assessment
FTE: Full-time equivalent
GMC: (GMC)
GP: General Practice/General Practitioner
GPNRO: General Practice National Recruitment Office
LTFT: Less than full-time training
MRCGP: Membership of the Royal College of General Practitioners
OOP: Out of Programme
RCGP: Royal College of General Practitioners
WPBA: Work-place Based Assessments (WPBAs)
